# Intrapartum Antibiotic Chemoprophylaxis Policies for the Prevention of Group B Streptococcal Disease Worldwide: Systematic Review

**DOI:** 10.1093/cid/cix654

**Published:** 2017-11-06

**Authors:** Kirsty Le Doare, Megan O’Driscoll, Kim Turner, Farah Seedat, Neal J Russell, Anna C Seale, Paul T Heath, Joy E Lawn, Carol J Baker, Linda Bartlett, Clare Cutland, Michael G Gravett, Margaret Ip, Shabir A Madhi, Craig E Rubens, Samir K Saha, Stephanie Schrag, Ajoke Sobanjo-ter Meulen, Johan Vekemans, Beate Kampmann, for the GBS Intrapartum Antibiotic Investigator Group, Angela Ramoni, Rikke Bek Helmig, Kaarin Makikallio, Tengiz Asatiani, Menachem Fisher, Michael Feinstein, Yuval Oz, Matan Elami Suzin, Vered Eisenberg, Alberto Berardi, Indi Trehan, Georgia Leigh Macad, Miha Lucovnik, Claire Nkiruka Oluwalana, Pippa Letchworth, Martin Jagoutz-Herzlinger, Francis Githae Muriithi, Kouther Issa Yassen, Gerard Visser, Sophie Cooper

**Affiliations:** 1 Centre for International Child Health, Imperial College London, United Kingdom; 2 Medical Research Council Unit, Fajara, The Gambia; 3 Vaccine Institute, Institute for Infection and Immunity, St George’s Hospital, University of London and St George’s University Hospitals NHS Foundation Trust, United Kingdom; 4 Global Medicine, University of Southern California, Los Angeles; 5 University of Warwick Medical School, Gibbet Hill, Coventry; 6 Maternal, Adolescent, Reproductive and Child Health Centre, London School of Hygiene & Tropical Medicine, United Kingdom; 7 King’s College London, United Kingdom; 8 College of Health and Medical Sciences, Haramaya University, Dire Dawa, Ethiopia; 9 Departments of Pediatrics and Molecular Virology and Microbiology, Baylor College of Medicine, Houston, Texas; 10 Department of International Health, Johns Hopkins Bloomberg School of Public Health, Baltimore, Maryland; 11 Medical Research Council: Respiratory and Meningeal Pathogens Research Unit, and Department of Science and Technology/National Research Foundation: Vaccine Preventable Diseases, University of the Witwatersrand, Faculty of Health Sciences, Johannesburg, South Africa; 12 Global Alliance to Prevent Prematurity and Stillbirth, Seattle, Washington; 13 Department of Obstetrics and Gynecology, University of Washington, Seattle; 14 Department of Microbiology, Faculty of Medicine, Chinese University of Hong Kong; 15 National Institute for Communicable Diseases, National Health Laboratory Service, Johannesburg, South Africa; 16 Department of Global Health, University of Washington, Seattle; 17 Bangladesh Institute of Child Health, Dhaka; 18 National Center for Immunization and Respiratory Diseases, Centers for Disease Control and Prevention, Atlanta, Georgia; 19 Bill & Melinda Gates Foundation, Seattle, Washington; 20 World Health Organization, Geneva, Switzerland; 21 Universitätsklinik, Innsbruck, Austria; 22 Department of Obstetrics and Gynaecology, Aarhus University Hospital, Skejby, Denmark; 23 Department of Obstetrics and Gynaecology, Turku University Hospital, Finland; 24 President of Georgian Obstetricians and Gynaecologists Association, State Medical University, Georgia; 25 Bar-Ilan Iniversity, Faculty of Medicine, Zefat, Israel; 26 Kaplan Hospital, Rechovot, Israel; 27 Israel; 28 Hadassah Hebrew University Medical Centre, Jerusalem, Israel; 29 MHA Sheba Medical Centre, Tel Hashomer and Sacker Faculty of Medicine, Tel Aviv, Israel; 30 Unit Unità Operativa di Terapia Intensiva Neonatale, Azienda Ospedaliero-Universitaria Policlinico, Modena, Italy; 31 Partners In Health, Liberia, Washington University, St Louis, Missouri; University of Malawi; 32 CPM (Certified Professional Midwife) Director and Founder of Abundant Grace of God Maternity Center, Tabuk City, Kalinga Philippines; 33 Department of Perinatology, Division of Obstetrics and Gynaecology, University Medical Centre, Ljubljana, Slovenia; 34 MRC Unit, The Gambia; 35 Chelsea and Westminster Hospital, London, UK; 36 practitioner, Austria; 37 Centre for Maternal and Newborn Health, Liverpool School of Tropical Medicine, Liverpool, UK; 38 Practitioner, Miassan, Iraq; 39 President International Federation of Obstetrics and Gynaecology, Geneva, Switzerland; 40 Royal College of Obstetrics and Gynaecology, London, UK

**Keywords:** group B *Streptococcus*, intrapartum antibiotic chemoprophylaxis

## Abstract

**Background:**

Intrapartum antibiotic chemoprophylaxis (IAP) prevents most early-onset group B streptococcal (GBS) disease. However, there is no description of how IAP is used around the world. This article is the sixth in a series estimating the burden of GBS disease. Here we aimed to review GBS screening policies and IAP implementation worldwide.

**Methods:**

We identified data through (1) systematic literature reviews (PubMed/Medline, Embase, Literature in the Health Sciences in Latin America and the Caribbean [LILACS], World Health Organization library database [WHOLIS], and Scopus) and unpublished data from professional societies and (2) an online survey and searches of policies from medical societies and professionals. We included data on whether an IAP policy was in use, and if so whether it was based on microbiological or clinical risk factors and how these were applied, as well as the estimated coverage (percentage of women receiving IAP where indicated).

**Results:**

We received policy information from 95 of 195 (49%) countries. Of these, 60 of 95 (63%) had an IAP policy; 35 of 60 (58%) used microbiological screening, 25 of 60 (42%) used clinical risk factors. Two of 15 (13%) low-income, 4 of 16 (25%) lower-middle–income, 14 of 20 (70%) upper-middle–income, and 40 of 44 (91%) high-income countries had any IAP policy. The remaining 35 of 95 (37%) had no national policy (25/33 from low-income and lower-middle–income countries). Coverage varied considerably; for microbiological screening, median coverage was 80% (range, 20%–95%); for clinical risk factor–based screening, coverage was 29% (range, 10%–50%). Although there were differences in the microbiological screening methods employed, the individual clinical risk factors used were similar.

**Conclusions:**

There is considerable heterogeneity in IAP screening policies and coverage worldwide. Alternative global strategies, such as maternal vaccination, are needed to enhance the scope of global prevention of GBS disease.

## Teaser

There is heterogeneity in intrapartum antibiotic use and implementation of policy varies widely. Global strategies to enhance the prevention of group B streptococcal disease that may include maternal vaccination are needed.

Group B *Streptococcus* (GBS; *Streptococcus agalactiae*) is a leading cause of early-onset disease in infants (EOGBS), defined as disease occurring on days 0–6 after birth [1]. Colonization of the maternal genitourinary or gastrointestinal tract [2] is a prerequisite for EOGBS disease [3, 4], with vertical transmission of GBS to babies occurring at or just before birth. The administration of intrapartum antibiotics aims to prevent EOGBS and is traditionally targeted based on known GBS colonization and/or the presence of peripartum clinical risk factors [4]. The potential mechanisms for prevention of EOGBS include reduction or suppression of maternal vaginal GBS colonization and thereby reduction of vertical transmission [5]. In addition, intrapartum antibiotic chemoprophylaxis (IAP) may allow the early treatment of GBS chorioamnionitis or fetal infection [6].

IAP has been recommended in the United States by the Centers for Disease Control and Prevention, American College of Obstetrics and Gynecologists, American Academy of Pediatrics, American Society for Microbiology, and American College of Nurse-Midwives since the early 1990s, with the first consensus policy in 1996 [7]. Initially the United States recommended both risk-based and microbiological screening. However, a large multicenter cohort study in 2002 [8] found microbiological screening to be superior in this setting. Subsequently, policy was changed to recommend microbiologic screening (using a rectovaginal swab) for GBS colonization at 35–37 weeks’ gestation or among women with threatened preterm delivery and unknown colonization status, and administration of high-dose intravenous benzylpenicillin or ampicillin in labor among those GBS colonized [4, 9]. Additionally, women with GBS bacteriuria or a previous infant with GBS disease as well as women with unknown colonization status and intrapartum risk factors such as prolonged rupture of membranes or maternal fever, are offered IAP in labor. A significant reduction in the incidence of EOGBS has been reported since the introduction of IAP policies [4, 9]; EOGBS disease in the United States declined from 1.7 per 1000 live births in the 1990s to 0.21 per 1000 live births in 2015 [10]. However, a recent Cochrane review found that although IAP may reduce the incidence of EOGBS, it did not result in a significant reduction in the mortality associated with EOGBS [11]. The review was critical of the quality of the studies included and considered there to be a high risk of bias in their methodology and execution.

Other countries such as the United Kingdom and the Netherlands have introduced IAP polices based on the presence of clinical risk factors. Risk factors used include preterm labor (<37 weeks) or premature or prolonged preterm rupture of membranes, GBS bacteriuria, previous infant with GBS disease, and maternal pyrexia (temperature >38°C) [12–14]. The use of clinical risk factor–based IAP strategies rather than microbiological screening is based on the assessment that the introduction of routine microbiological screening may not be cost-effective and that clinical risk factor–based IAP may result in fewer women being exposed to the potential risks associated with widespread antibiotic use [14, 15]. There is currently no international consensus as to whether IAP is best achieved through microbiological screening or based on the presence of clinical risk factors. There is evidence from the United States that the incidence of EOGBS has declined since the introduction of clinical screening strategies, although a larger proportion of women are treated with IAP using clinical as opposed to risk-based strategies [8]. Any consensus regarding IAP strategies should take potentially opposing points into consideration.

**Figure 1.**
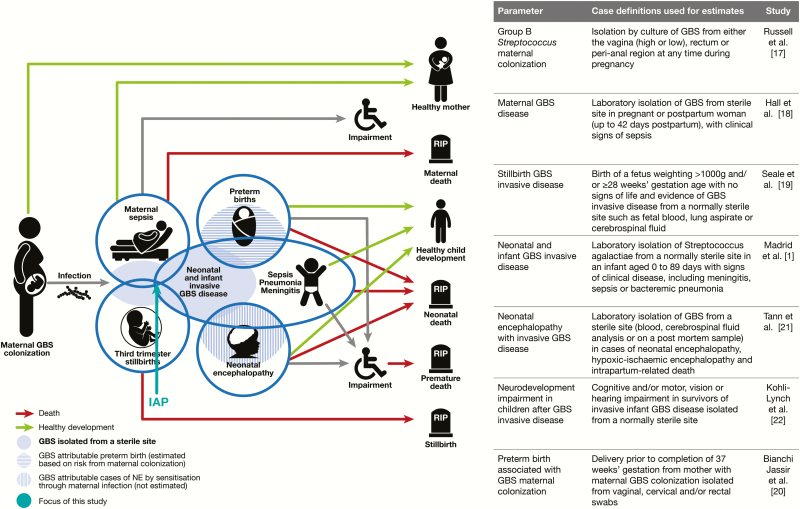
Intrapartum antibiotic chemoprophylaxis in disease schema for group B streptococcal (GBS) disease, as described by Lawn et al [16].

This review is part of a supplement estimating the burden of GBS disease in pregnant women, stillbirths, and infants, an important topic that has important implications for public health policy as well as for future vaccine development [16]. The supplement includes systematic reviews and meta-analyses on GBS colonization, and adverse outcomes associated with GBS around birth [1, 2, 17–22], which form input parameters to a compartmental model (Figure 1) [23]. These are reported individually and according to international guidelines [24, 25].

The objectives of this review are:

1. To undertake a comprehensive and systematic literature review, and a survey of national obstetric and gynecological societies, to assess the presence of IAP policies worldwide, the strategies and methods used, and where possible, coverage or the status of implementation;
2. To assess these data for input into estimating the global burden of GBS in pregnancy, stillbirth, and infants;
3. To summarize the data gaps to inform future strategies for the prevention of GBS disease, including maternal vaccination, globally.

## METHODS

This article is part of a protocol entitled “Systematic estimates of the global burden of Group B *Streptococcus* in pregnant women, stillbirths and infants,” which was submitted for ethical approval to the London School of Hygiene & Tropical Medicine (reference number 11966) and approved on 30 November 2016.

### Data Searches and Inputs

We identified data through 2 sources: (1) systematic review of the published literature and (2) reviews of online policies and an online survey submitted to clinicians, researchers, and relevant professionals worldwide.

### Literature Searches

We undertook systematic literature searches of Medline, Embase, Literature in the Health Sciences in Latin America and the Caribbean (LILACS), Scopus, and the World Health Organization library database (WHOLIS). The literature search was updated on 20 January 2017. We used the search terms “antibiotics,” “intrapartum,” “group B *Streptococcus*,” and “colonization” with no date or language restrictions (full search terms are listed in Supplementary Table 1). We additionally searched the China Academic Journals Full-Text Database (with a time restriction of 3 years), and a Russian online database (Cyberleninka) with no date restrictions, as these data are often not present on classical database searches. We abstracted the data from articles in foreign languages alongside translators where available and used automatic translation if native speakers were unavailable.

### Reviews of Online Policies and an Online Survey

We searched for online policies of all 130 countries listed on the International Federation of Gynecology and Obstetrics (FIGO) website [26] to identify the latest policies. In addition, we designed an online survey (Supplementary Materials 2) and approached the World Health Organization (WHO) Safer Childbirth Group, FIGO, the Royal College of Obstetricians and Gynaecologists, and the European Society for Paediatric Infectious Diseases to disseminate to their members.

### Inclusion and Exclusion Criteria

Two authors (K. L. D. and K. T.) independently abstracted data onto standard Excel data entry forms. Policies were reviewed according to the inclusion and exclusion criteria below. For any discrepancies, a third researcher (M. O.) was consulted. We included any document detailing IAP policy and strategy: microbiological screening (including sample site of swab and gestation of screening), clinical risk factors, the type and route of antibiotic administered, and an estimate of IAP coverage within that country. We excluded any policy that had been revoked or was under review (Supplementary Table 2).

To ensure results were current, we selected the latest available policy from each country. The hierarchy of policy documents, based on their likely accuracy, was as follows: (1) national policy document available from relevant national association; (2) national policy available from published literature; (3) national policy available from survey participant.

We categorized countries into 4 groups: (1) no IAP policy or low implementation (0%); (2) microbiological screening policy with limited implementation (defined as <50% of eligible women receiving IAP); (3) clinical risk factor–based screening with high implementation (defined as >50% of eligible women receiving IAP); (4) universal microbiological screening policy with high implementation (defined as ≥50% of eligible women receiving IAP). We did an ecological analysis comparing country IAP policy by category with EOGBS disease incidence as a scatterplot.

## RESULTS

### Study Selection

**Figure 2.**
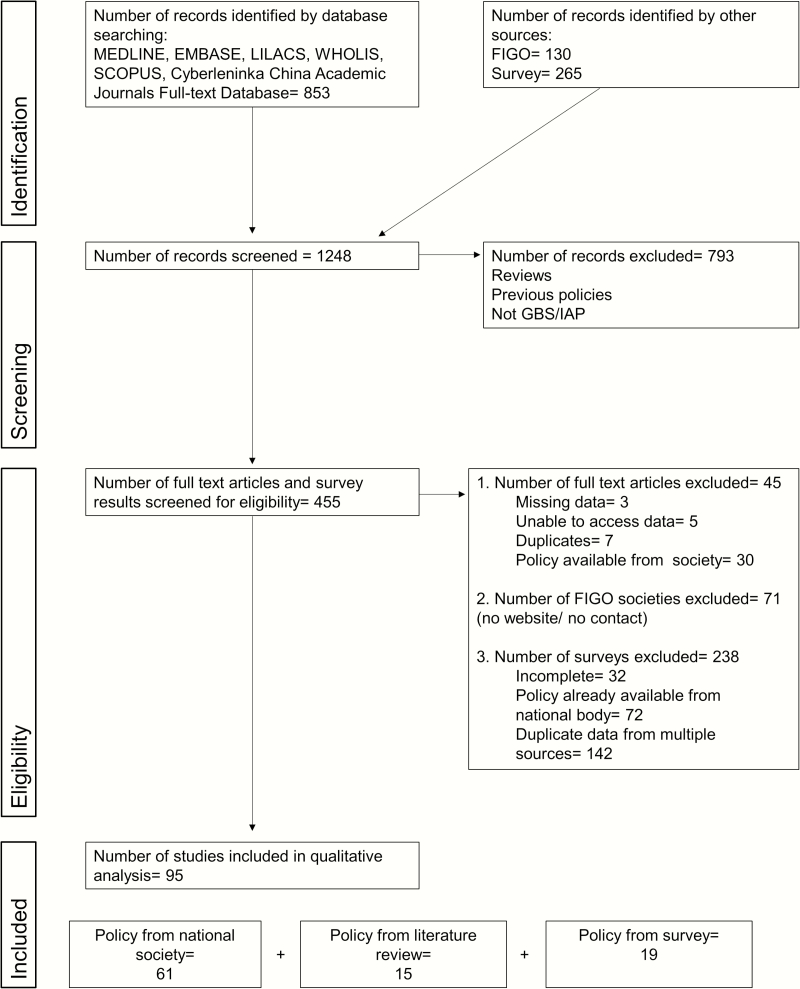
Flowchart of intrapartum antibiotic chemoprophylaxis policy data identified through systematic searches and other means. Abbreviations: FIGO, International Federation of Gynecology and Obstetrics; GBS, group B *Streptococcus*; IAP, intrapartum antibiotic chemoprophylaxis; PRISMA, Preferred Reporting Items for Systematic Reviews and Meta-analyses.

We identified 853 articles, of which we retained 60 after title and abstract screening for review of full texts (Figure 2). We excluded a further 15 articles after full-text review as these either presented duplicate data (n = 7), did not have policy data in the main text (n = 3), or did not make full text available (n = 5), leaving 45 articles for inclusion in the analysis. Of these, details of policy were already available from 30 national policy documents, leaving 15 articles containing additional policy information.

Using the international list of obstetric and gynecological societies listed on the FIGO website, we identified online policy documents from 42 countries and received responses that there was no national policy from 19 countries through FIGO. Of the remaining 71 countries, either there was no society website or no working email address, or the contacts did not reply to the FIGO email, despite receiving a read-receipt.

We received responses from 265 participants to our survey (Figure 2). We excluded 238 due to duplicate entries with identical responses from participants in the same country (n = 142), incomplete responses (n = 32), or because policy documents had already been received from the country’s obstetric association and survey responses matched the country policy (n = 72), leaving 19 unique survey responses. Details are reported in Supplementary Table 3. The final dataset included policies from 95 countries (FIGO = 61; original articles = 15; survey = 19)

### Intrapartum Antibiotic Chemoprophylaxis Policy and Implementation Strategies

**Figure 3.**
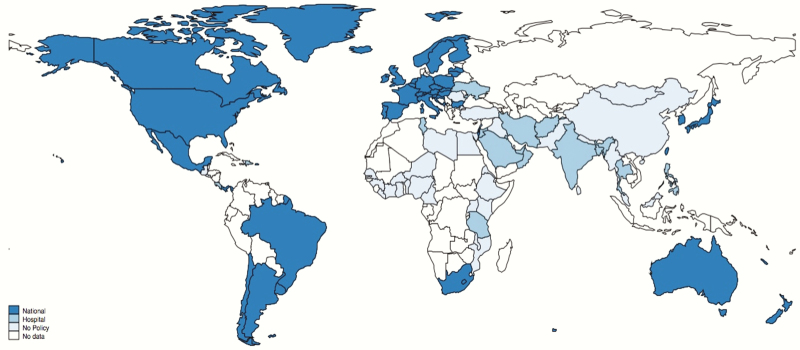
Distribution of national policies for maternal group B *Streptococcus* screening and administration of intrapartum antibiotics. Borders of countries/territories in map do not imply any political statement.

**Figure 4.**
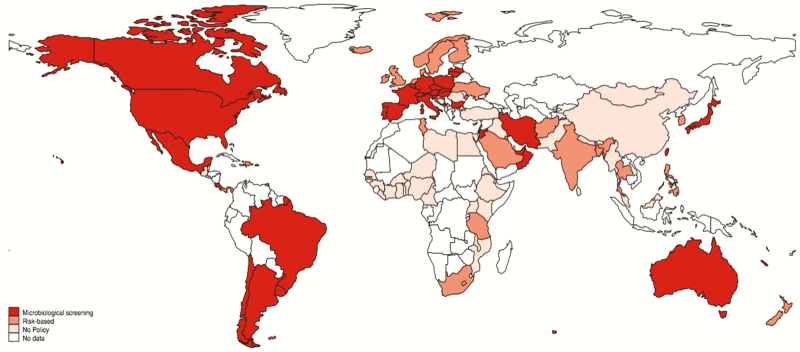
Distribution of policies for maternal group B *Streptococcus* screening by type of policy. Borders of countries/territories in map do not imply any political statement.

Sixty of 95 countries had a national IAP policy. The strategies used varied: 25 of 60 (42%) used a risk factor–based approach and 35 of 60 (58%) used both microbiological screening and risk factor approaches. Thirty-five of 95 (37%) countries had no national policy. Within these, there were reports of policies from hospitals or localities in 9 countries. Of these, 5 of 35 used rapid screening at point of labor only and 4 of 35 used risk factor approaches. Figure 3 shows countries where IAP policies were identified and Figure 4 shows IAP policy type globally.

### Intrapartum Antibiotic Chemoprophylaxis Policy by Region and Income Status

We received responses concerning IAP policy from all regions. All developed region countries had an IAP policy (34/34). Other regions varied with the majority of countries in sub-Saharan Africa (3/20) and East Asia (1/3) reporting no IAP policy (Table 1).

**Table 1.** Intrapartum Antibiotic Chemoprophylaxis by Region and Income Status

Region	Responses	IAP Policy	Microbiological	Risk Factor Based	No Policy
Developed regions	34	34	22	12	0
East Asia	3	1	0	1	2
Latin America	12	9	7	2	3
North Africa	3	1	0	1	2
Southeast Asia	7	5	3	2	4
South Asia	7	4	1	2	3
Sub-Saharan Africa	20	3	0	2	17
West Asia	9	5	2	3	4

Abbreviation: IAP, intrapartum antibiotic chemoprophylaxis.

The majority of countries reporting no policy came from low and lower-middle–income countries (25/33 [76%]). Two countries had limited IAP strategies, reported as risk-based screening in hospitals run by the charitable organization Médecins sans Frontières. Of countries reporting any IAP policy, 4 of 16 (25%) were from lower-middle–income countries (all risk-based screening policies). In upper-middle–income countries, 8 of 20 reported microbiological policy and 6 of 20 risk-based policy, whereas in high-income countries, 24 of 44 had microbiological and 16 of 44 risk-based policies. (Supplementary Table 3).

For countries with an IAP policy, implementation varied both within and between countries. Implementation was more frequently reported as high in countries with microbiological screening (median, 80% [range, 20%–95%]) compared to countries with clinical risk factor–based approaches (29% [range, 10%–50%]). Policy documents estimating implementation varied from estimates based on clinician reporting, especially in countries using clinical risk factor–based approaches, where clinician reporting was lower than that reported in policy documents.

### Microbiological Screening for Intrapartum Antibiotic Chemoprophylaxis Administration

In 35 countries reporting microbiological screening (30 national screening, 5 hospital-level screening), women were usually screened at 35–37 weeks’ gestation. Two countries (Bulgaria and Japan) offered additional microbiological screening at 20 weeks’ gestation. In 5 countries (Poland, Bangladesh, Iran, Thailand, and Trinidad and Tobago), individual hospitals offered point-of-care screening using polymerase chain reaction when women presented in labor, in addition to screening at 35–37 weeks’ gestation. The estimated coverage of these guidelines varied greatly and ranged from 20% in Brazil to 89% in the United States and Belgium (median, 80%). Where microbiological screening methods were reported (n = 35), 21 reported sampling both the rectum and vagina either with separate (n = 2) or combined (n = 19) swabs, and 14 used only vaginal swabs.

### Clinical Risk Factors for Intrapartum Antibiotic Chemoprophylaxis Administration

Twenty-five countries reported IAP based on clinical risk factors (14 national, 11 regional/hospital). There was little variation in risk factors used to determine the need for IAP. All recommended IAP use if a previous infant had GBS disease; the majority (23/25) also recommended IAP for preterm prolonged rupture of membranes, premature rupture of membranes >18 hours (PROM), or maternal GBS bacteriuria. The clinical risk factors are listed in Table 2.

**Table 2.** Clinical Risk Factors Used as a Basis for Antibiotic Administration to Reduce Group B Streptococcal Disease at Delivery

Risk Factor	No. of Countries Reporting (n = 25)
Previous infant with GBS	25
GBS in urine	23
PROM >18 h	23
PROM >24 h	2
Premature labor	23
Maternal fever	20
Chorioamnionitis	2

Abbreviations: GBS, group B Streptococcus; PROM, premature rupture of membranes.

Two middle-income countries reported IAP administration based on clinical risk factors (Kenya and South Africa). However, responses from the online survey suggest that coverage of this policy in these countries was low.

### Clinician Responses to Survey Compared to National Policy

There was considerable variation in the survey responses between countries concerning the question “Does your country have a national policy for intrapartum antibiotics to prevent neonatal group B streptococcal disease?” In countries with risk-based screening policies, one-third of respondents answered “no,” although their obstetric societies have published national guidance. There was no discrepancy in countries reporting microbiological screening approaches.

### Antibiotics Administered

The majority of policies (50/60) recommended intravenous penicillin–based antibiotics (38/50 penicillin, 12/50 ampicillin) and clindamycin in cases of confirmed penicillin allergy. Six countries recommended a cephalosporin rather than penicillin and 4 countries in South America and 2 in Asia recommended additional vancomycin because of concerns about a theoretical risk of developing antibiotic resistance in their populations in patients with penicillin allergy.

### Effect of Screening Policy on EOGBS Incidence in Countries Reporting Both National Policy and National EOGBS Incidence

**Figure 5.**
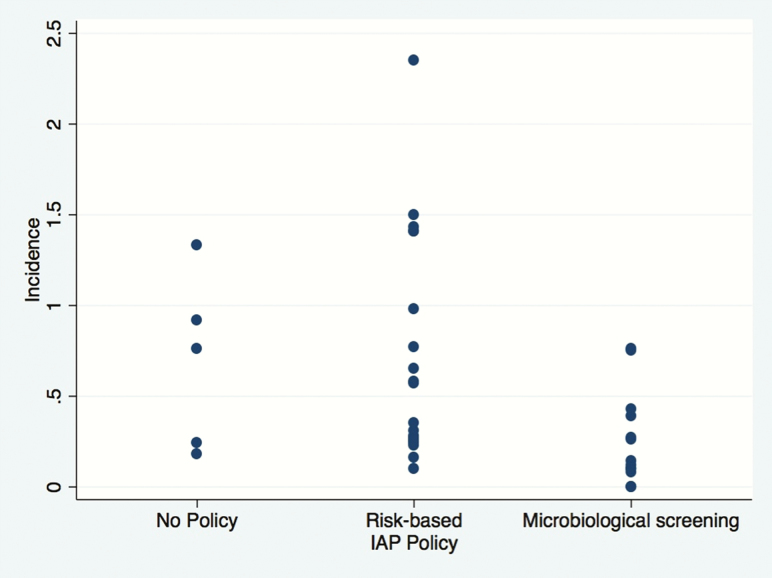
Scatterplot of early-onset disease incidence by national intrapartum antibiotic chemoprophylaxis (IAP) policy type.

EOGBS incidence data were available for 32 countries: 12 reporting a microbiological and risk-based strategy, 15 reporting a clinical risk factor strategy, and 5 reporting no national policy. The results are presented in Figure 5. Broadly, EOGBS incidence was lower in countries with a microbiological and clinical risk–based policy [1].

## DISCUSSION

This review of IAP policies to prevent EOGBS disease represents the first systematic review and survey of GBS screening policies to date, with data from 95 countries of the 195 United Nations member states. It clearly demonstrates that IAP policy, strategy, and implementation are heterogeneous and different disease burden estimates and healthcare systems have led to a range of IAP approaches. This may imply that opportunities for prevention of EOGBS are being missed in some settings.

Whereas the WHO advocates screening for GBS during pregnancy, it also recognizes that screening for all pregnant women—especially in settings with known low maternal colonization prevalence, low-resource countries, and/or countries where provision of care is limited—is difficult to implement. The WHO therefore recommends that IAP should be implemented within the context of local policy and guidance on screening for GBS colonization [27]. In our study, low- and middle-income countries more frequently reported having no IAP policy as compared to high-income countries. There were exceptions to this; for example, both Kenya and South Africa had national IAP policies, although the survey suggested that implementation and coverage were low. In countries where access to skilled care is limited, or where the majority of births occur at home (eg, in some low-income countries where up to 80% of births are at home), implementation of IAP will always pose a challenge.

Other concerns relate to the acceptability of screening by women in high-income countries, especially the use of vaginal and rectal swabs and increased interventions during pregnancy that will limit the implementation of any IAP strategy.

It is estimated that >40% of women in the United States now receive antibiotics in labor, either for the prevention of EOGBS disease or to prevent postpartum infections following cesarean delivery [28]. Such widespread use of antibiotics in pregnancy has been raised as a concern, both in terms of potential impact on antimicrobial resistance and on possible long-term consequences in infants exposed in utero or around the time of birth. For example, an increase in infections due to *Escherichia coli*, especially in preterm infants, was demonstrated, following widespread IAP use in the United States [29, 30]. However, despite almost 20 years of IAP use, there is no evidence for this in large epidemiological studies in the United States [31]. Other issues include maternal anaphylaxis to β-lactams [32]. More theoretical concerns include the effect of maternal antibiotic use on the maternal and infant gut and skin flora, on the neonatal microbiome, and on subsequent immunological priming [33]. Alterations in the neonatal intestinal microbiome have been associated with increased rates of allergy, asthma, and obesity [34]. This is an area that requires further investigation in countries that offer IAP.

A maternal GBS vaccine may be the ideal solution to help reduce the burden of neonatal GBS disease in all settings. Implementation of a vaccine is likely to be higher than successful administration of IAP, especially in low- and middle-income settings and will protect against at least 75% of EOGBS. However, in some high-income settings, it may be feasible to combine with IAP when vaccination might be unavailable or suboptimal [35].

Our study also provides important additional information on national policies through its searches outside of scientific literature. However, responses from individual clinicians may not necessarily be representative of the different levels of healthcare and of health facilities that exist within a country, and we acknowledge the limitations to this approach. In high-income contexts, countries using both microbiological and clinical risk factor–based approaches for IAP had low reported EOGBS incidence [1]. We cannot assume causality, however, as this finding may reflect other differences in healthcare such as access to care. We were also unable to correlate reported EOGBS incidence with the introduction of IAP policy in all of these countries, as policy may have been put into place after EOGBS data were collected. The reliability of data on implementation coverage of policies are hard to verify, and we have therefore categorized them using very broad thresholds (< 50% or >50%). This is important in terms of understanding the burden of disease; the category influences the risk of EOGBS disease [2] at an individual level, and is thus important in terms of a compartmental approach to modeling disease burden [16].

In conclusion, local, national, and international policies should be informed by the strongest evidence for the prevention of EOGBS and might require tailored solutions to succeed. Future GBS vaccines may offer the best opportunity to prevent EOGBS in any setting, with better implementation than policies for IAP currently in use (Table 3).

**Table 3.** Key Findings and Implications

What’s new about this? • GBS is an important perinatal pathogen. IAP is the most widely practiced intervention for preventing early onset GBS, yet no worldwide data exists regarding national policies or levels of implementation. This is the first systematic review of IAP policies based on published data, online survey, and reviews of national policies.
What was the main finding? • Data were identified on IAP policy for 95 countries (of 195 UN member states). Content on policy approach by country included microbiological screening and which antibiotics used.
How can the data be improved? • There is no current means to routinely track GBS IAP policy at the national level, and it would be beneficial to include in the WHO annual Maternal Neonatal and Child Health policy survey. Data for implementation/coverage are even harder to identify; therefore, it is difficult to assess program status or effect.
What does it mean for policy and programs? • Variable policies—and in some countries internal inconsistencies in reporting of policies, and likely even more variable implementation between and within countries. These data are relevant in considering maternal GBS vaccination—which may be more likely to reach the poorest at higher coverage than IAP and also be easier to track in national policy.

Abbreviations: GBS, group B Streptococcus; IAP, intrapartum antibiotic chemoprophylaxis; UN, United Nations; WHO, World Health Organization.

## Supplementary Data

Supplementary materials are available at *Clinical Infectious Diseases* online. Consisting of data provided by the authors to benefit the reader, the posted materials are not copyedited and are the sole responsibility of the authors, so questions or comments should be addressed to the corresponding author.

## Supplementary Material

Supplement material 1

Supplement material 2

## Notes

***Author contributions.*** The concept of the estimates and the technical oversight of the series were led by J. E. L. and A. C. S. The reviews, analyses, and first draft of the manuscript were undertaken by K. L. D. with K. T., F. S., M. O., B. K., A. C. S., J. E. L., and P. T. H. Survey design was undertaken by K. L. D. and K. T. The GBS Estimates Expert Advisory Group (C. J. B., L. B., C. C., M. G. G., M. I., S. A. M., C. E. R., S. K. S., S. S., A. S.-t. M., J. V.) contributed to the conceptual process throughout, notably on the disease schema and data inputs. The IAP investigator group (Angela Ramoni, Rikke Bek Helmig, Kaarin Makikallio, Tengiz Asatinai, Menachem Fisher, Michael Feinstein, Oz Yuval, Matan Elami Suzin, Vered Eisenberg, Alberto Berardi, Indi Trehan, Georgia Leigh Macad, Miha Lucovnik, Claire Nkiruka Oluwalana, Pippa Letchworth, Martin Jagoutz-Herlinger, Francis Githae Muriithi, Kouther Issa Yassen, Gerard Visser, Johan Vos, and Sophie Cooper) input data for the analyses. All the authors reviewed and gave input to the manuscript.

***Acknowledgments.*** We thank Jane Plumb, Group B Strep Support, Dr Tejshri Shah, Médecins sans Frontières (MSF), and Anne Cugier, MSF, for helping with the dissemination of the survey; Claudia da Silva for administrative assistance; and Alegria Perez for coordinating author signatures.

***GBS Intrapartum Antibiotic Investigator Group.*** Dr Angela Ramoni (Universitätsklinik, Innsbruck, Austria), Dr Rikke Bek Helmig (Department of Obstetrics and Gynaecology, Aarhus University Hospital, Skejby, Denmark), Dr Kaarin Makikallio (Department of Obstetrics and Gynaecology, Turku University Hospital, Finland), Prof. Tengiz Asatiani (President of Georgian Obstetricians and Gynaecologists Association, State Medical University, Georgia), Dr Menachem Fisher (Bar-Ilan Iniversity, Faculty of Medicine, Zefat, Israel), Dr Michael Feinstein (Kaplan Hospital, Rechovot, Israel), Dr Yuval Oz (Israel), Dr Matan Elami Suzin (Hadassah Hebrew University Medical Centre, Jerusalem, Israel), Dr Vered Eisenberg (MHA Sheba Medical Centre, Tel Hashomer and Sacker Faculty of Medicine, Tel Aviv, Israel), Dr Alberto Berardi (Unit Unità Operativa di Terapia Intensiva Neonatale, Azienda Ospedaliero-Universitaria Policlinico, Modena, Italy), Dr. Indi Trehan (Partners In Health, Liberia, Washington University, St Louis, Missouri; University of Malawi), Mrs. Georgia Leigh Macad (CPM (Certified Professional Midwife) Director and Founder of Abundant Grace of God Maternity Center, Tabuk City, Kalinga Philippines), Assoc. Prof. Miha Lucovnik (Department of Perinatology, Division of Obstetrics and Gynaecology, University Medical Centre, Ljubljana, Slovenia), Dr Claire Nkiruka Oluwalana (MRC Unit, The Gambia), Dr. Pippa Letchworth (Chelsea and Westminster Hospital, London, UK), Dr. Martin Jagoutz-Herzlinger (practitioner, Austria), Dr. Francis Githae Muriithi (Centre for Maternal and Newborn Health, Liverpool School of Tropical Medicine, Liverpool, UK), Dr. Kouther Issa Yassen (Practitioner, Miassan, Iraq), Prof. Gerard Visser (President International Federation of Obstetrics and Gynaecology, Geneva, Switzerland), Ms. Sophie Cooper (Royal College of Obstetrics and Gynaecology, London, UK).

***Financial support.*** This supplement was supported by a grant to the London School of Hygiene & Tropical Medicine from the Bill & Melinda Gates Foundation (reference number OPP1131158).

Supplement sponsorship. This article appears as part of the supplement “The Burden of Group B *Streptococcus* Worldwide for Pregnant Women, Stillbirths, and Children,” sponsored by the Bill & Melinda Gates Foundation and coordinated by the London School of Hygiene & Tropical Medicine.

***Potential conflicts of interest.*** Many contributors to this supplement have received funding for their research from foundations, especially the Bill & Melinda Gates Foundation, and several from Wellcome Trust, Medical Research Council UK, the Thrasher Foundation, the Meningitis Research Foundation, and one individual from the US National Institutes of Health. Members of the Expert Advisory Group received reimbursement for travel expenses to attend working meetings related to this series. A. S.-t. M. works for the Bill & Melinda Gates Foundation. A. B. attended a Pfizer Advisory Board meeting in Paris, 2015; received consultancy funding from Putnam Associates; received a consultancy fee from GSK for a conference interview; and collaborated in the Devani Project funded by the European Union. B. K. has participated in board meetings as scientific advisor for GSK and Pfizer on the subject of maternal immunization. C. J. B. has served as a member of the Presidential Advisory Committee for Seqirus Inc and of the CureVac Inc Scientific Advisory Committee, as well as undertaken consultancy work for Pfizer Inc. C. C. has received institutional compensation from Novartis for conducting GBS studies. P. T. H. has been a consultant to Novartis and Pfizer on GBS vaccines but received no funding for these activities. M. I. has undertaken sponsored research from Pfizer on pneumococcal disease in adults and from Belpharma Eumedica (Belgium) on temocillin antimicrobial susceptibility in Enterobacteriaceae. K. L. D. has received funding by the Bill & Melinda Gates Foundation to work on research on GBS serocorrelates of protection to inform vaccine trials, and travel expenses from Pfizer to attend a meeting on an investigator-led project on GBS. S. A. M. has collaborated on GBS grants funded by GlaxoSmithKline and by Pfizer and received personal fees for being member of its advisory committee; he has also collaborated on a GBS grant funded by Minervax. All other authors report no potential conflicts of interest. All authors have submitted the ICMJE Form for Disclosure of Potential Conflicts of Interest. Conflicts that the editors consider relevant to the content of the manuscript have been disclosed.

## References

[1] Madrid L, Seale AC, Kohli-Lynch M Infant group B streptococcal disease incidence and serotypes worldwide: systematic review and meta-analyses. Clin Infect Dis 2017; 65(suppl 2):S160–72. 29117326 10.1093/cid/cix656 PMC5850457

[2] Russell N, Seale AC, O’Sullivan C Risk of early-onset neonatal group B streptococcal disease with maternal colonization worldwide: systematic review and meta-analyses. Clin Infect Dis 2017; 65(suppl 2):S152–9. 29117325 10.1093/cid/cix655 PMC5850448

[3] Schrag SJ, Verani JR Intrapartum antibiotic prophylaxis for the prevention of perinatal group B streptococcal disease: experience in the United States and implications for a potential group B streptococcal vaccine. Vaccine 2013; 31 (suppl 4):D20–6. 23219695 10.1016/j.vaccine.2012.11.056 PMC11843781

[4] Verani JR, McGee L, Schrag SJ; Division of Bacterial Diseases, National Center for Immunization and Respiratory Diseases, Centers for Disease Control and Prevention (CDC) Prevention of perinatal group B streptococcal disease—revised guidelines from CDC, 2010. MMWR Recomm Rep 2010; 59:1–36. 21088663

[5] Baltimore RS Consequences of prophylaxis for group B streptococcal infections of the neonate. Semin Perinatol 2007; 31:33–8. 17317425 10.1053/j.semperi.2007.01.005

[6] Martinelli P, Sarno L, Maruotti GM, Paludetto R Chorioamnionitis and prematurity: a critical review. J Matern Fetal Neonatal Med 2012; 25(suppl 4):29–31. 22958008 10.3109/14767058.2012.714981

[7] ACOG Committee Opinion. Prevention of early-onset group B streptococcal disease in newborns. Number 173—June 1996. Committee on Obstetric Practice. American College of Obstetrics and Gynecologists. Int J Gynaecol Obstet 1996; 54: 197–205. 9236325

[8] Schrag S, Gorwitz R, Fultz-Butts K, Schuchat A Prevention of perinatal group B streptococcal disease. Revised guidelines from CDC. MMWR Recomm Rep 2002; 51:1–22. 12211284

[9] Colbourn T, Gilbert R An overview of the natural history of early onset group B streptococcal disease in the UK. Early Hum Dev 2007; 83:149–56. 17300884 10.1016/j.earlhumdev.2007.01.004

[10] Centers for Disease Control and Prevention. Active Bacterial Core Surveillance (ABCs) Report. Emerging Infections Program Network Group B Streptococcus, 2015. Atlanta, 2015.

[11] Ohlsson A, Shah VS Intrapartum antibiotics for known maternal group B streptococcal colonization. Cochrane Database Syst Rev 2014; CD007467. 24915629 10.1002/14651858.CD007467.pub4

[12] Royal College of Obstetricians and Gynaecologists. The prevention of early onset neonatal group B streptococcal disease. London: RCOG, 2012.

[13] Nederlandse Vereniging voor Obstetrie en Gynaecologie. Preventie van neonatale groep B streptokokken [in Dutch]. Amsterdam: Nederlandse Vereniging voor Obstetrie en Gynaecologie, 2008.

[14] Royal College of Obstetricians and Gynaecologists. Audit of current practice in preventing early-onset neonatal group B streptococcal disease in the UK. London: RCOG, 2012.

[15] Kolkman DG, Rijnders ME, Wouters MG Implementation of a cost-effective strategy to prevent neonatal early-onset group B haemolytic *Streptococcus* disease in the Netherlands. BMC Pregnancy Childbirth 2013; 13:155. 23899463 10.1186/1471-2393-13-155 PMC3733882

[16] Lawn JE, Bianchi-Jassir F, Russell 1Group B streptococcal disease worldwide for pregnant women, stillbirths, and children: why, what and how to undertake estimates? Clin Infect Dis 2017; 65(suppl 2):S89–100. 29117323 10.1093/cid/cix653 PMC5850012

[17] Russell N, Seale AC, O’Driscoll M Maternal colonization with group B *Streptococcus* and serotype distribution worldwide: systematic review and meta-analyses. Clin Infect Dis 2017; 65(suppl 2):S100–11. 29117327 10.1093/cid/cix658 PMC5848259

[18] Hall J, Hack Adams N, Bartlett L Maternal disease with group B *Streptococcus* and serotype distribution worldwide: systematic review and meta-analyses. Clin Infect Dis 2017; 65(suppl 2):S112–24. 29117328 10.1093/cid/cix660 PMC5850000

[19] Seale AC, Blencowe H, Bianchi-Jassir F Stillbirth with group B streptococcal disease worldwide: systematic review and meta-analyses. Clin Infect Dis 2017; 65(suppl 2):S125–32. 29117322 10.1093/cid/cix585 PMC5850020

[20] Bianchi-Jassir F, Seale AC, Kohli-Lynch M Preterm birth associated with group B *Streptococcus* maternal colonization worldwide: systematic review and meta-analyses. Clin Infect Dis 2017; 65(suppl 2):S133–42. 29117329 10.1093/cid/cix661 PMC5850429

[21] Tann CJ, Martinello K, Sadoo S Neonatal encephalopathy with group B streptococcal disease worldwide: systematic review, investigator group datasets, and meta-analysis. Clin Infect Dis 2017; 65(suppl 2):S173–89. 29117330 10.1093/cid/cix662 PMC5850525

[22] Kohli-Lynch M, Russell N, Seale AC Neurodevelopmental impairment in children after group B streptococcal disease worldwide: systematic review and meta-analyses. Clin Infect Dis 2017; 65(suppl 2):S190–9. 29117331 10.1093/cid/cix663 PMC5848372

[23] Seale AC, Bianchi-Jassir F, Russell N Estimates of the burden of group B streptococcal disease worldwide for pregnant women, stillbirths, and children. Clin Infect Dis 2017; 65(suppl 2):S200–19. 29117332 10.1093/cid/cix664 PMC5849940

[24] Liberati A, Altman DG, Tetzlaff J The PRISMA statement for reporting systematic reviews and meta-analyses of studies that evaluate health care interventions: explanation and elaboration. PLoS Med 2009; 6:e1000100. 19621070 10.1371/journal.pmed.1000100 PMC2707010

[25] Stevens GA, Alkema L, Black RE GATHER Working Group Guidelines for accurate and transparent health estimates reporting: the GATHER statement. PLoS Med 2016; 13:e1002056. 27351744 10.1371/journal.pmed.1002056 PMC4924581

[26] International Federation of Gynecology and Obstetrics. Our members Available at: http://www.figo.org/our-members. Accessed 25 January 2017.

[27] World Health Organization. WHO recommendations for prevention and treatment of maternal peripartum infections. Geneva, Switzerland: WHO, 2015. 26598777

[28] Ledger WJ, Blaser MJ Are we using too many antibiotics during pregnancy? BJOG 2013; 120:1450–2. 24118809 10.1111/1471-0528.12371 PMC4492536

[29] Bizzarro MJ, Dembry LM, Baltimore RS, Gallagher PG Changing patterns in neonatal *Escherichia coli* sepsis and ampicillin resistance in the era of intrapartum antibiotic prophylaxis. Pediatrics 2008; 121:689–96. 18381532 10.1542/peds.2007-2171

[30] Schrag SJ, Hadler JL, Arnold KE, Martell-Cleary P, Reingold A, Schuchat A Risk factors for invasive, early-onset *Escherichia coli* infections in the era of widespread intrapartum antibiotic use. Pediatrics 2006; 118:570–6. 16882809 10.1542/peds.2005-3083

[31] Schrag SJ, Farley MM, Petit S Epidemiology of invasive early-onset neonatal sepsis, 2005 to 2014. Pediatrics 2016; 138. 10.1542/peds.2016-2013 27940705

[32] Mulla ZD, Ebrahim MS, Gonzalez JL Anaphylaxis in the obstetric patient: analysis of a statewide hospital discharge database. Ann Allergy Asthma Immunol 2010; 104:55–9. 20143646 10.1016/j.anai.2009.11.005

[33] Cacho N, Neu J Manipulation of the intestinal microbiome in newborn infants. Adv Nutr 2014; 5:114–8. 24425730 10.3945/an.113.004820 PMC3884092

[34] Castanys-Muñoz E, Martin MJ, Vazquez E Building a beneficial microbiome from birth. Adv Nutr 2016; 7:323–30. 26980815 10.3945/an.115.010694 PMC4785476

[35] Kim SY, Russell LB, Park J Cost-effectiveness of a potential group B streptococcal vaccine program for pregnant women in South Africa. Vaccine 2014; 32:1954–63. 24530145 10.1016/j.vaccine.2014.01.062

