# Supplementary material for: Intrapartum Antibiotic Chemoprophylaxis Policies for the Prevention of Group B Streptococcal Disease Worldwide: Systematic Review
Source: Clin Infect Dis. 2017 Nov 6;65(Suppl 2):S143–51. doi: 10.1093/cid/cix654 (PMC5850619; doi:10.1093/cid/cix654)
Supplement: Supplement material 1 [file cix654_suppl_supplement-material1.pdf]

The burden of Group B *Streptococcus* worldwide for pregnant women, stillbirths and children

Paper 6: Intrapartum antibiotic chemoprophylaxis policies for the prevention of Group B Streptococcal Disease worldwide: systematic review

Supplementary information

Contents

Systematic estimates of the burden of Group B *Streptococcus* worldwide in pregnant women, stillbirths and infants 1

Paper 6: Intrapartum antibiotic chemoprophylaxis policies for the prevention of Group B Streptococcal Disease worldwide: systematic review 1

Supplementary information 1

Supplementary Table S1: search terms 3

Supplementary Table S2: inclusion and exclusion criteria 4

Supplementary Table S3: Study characteristics and data 5

References 10

Supplementary Table S1: search terms

(“Pregnant women” [MESH Terms], OR “Pregnancy” [MESH Terms], OR “pregnan*” [All Fields] OR “Matern”* [All Fields], OR “pregnan*” [All Fields] OR “intrapartum” [All fields] OR “coloni*” [All fields] OR “Antibiotic Prophylaxis” [MESH Terms] OR “Anti infective agents” [MESH Terms] OR “antibiot*” [All fields].

AND

(“Streptococcus” [All Fields] OR “Streptococcal” [All Fields] OR “Streptococci” [All Fields]) AND ((“Group” AND “B”) or “Agalactiae”) OR “Streptococcus Agalactiae” [MeSH Terms]

Limit: humans

Supplementary Table S2: inclusion and exclusion criteria

	Inclusion criteria	Exclusion criteria
**National Society Policy**	Published Society IAP policy and strategy: microbiological screening (including sample site of swab and gestation of screening), clinical risk factors, type and route of antibiotic.	Policies revoked or under review.
**Literature Search**	Published Society IAP policy and strategy: microbiological screening (including sample site of swab and gestation of screening), clinical risk factors, type and route of antibiotic.	Policy already found from national society, duplicate policy or policy not defined in methods.
**Online Survey**	Clinician responses of IAP policy and strategy: microbiological screening (including sample site of swab and gestation of screening), clinical risk factors, type and route of antibiotic.	Duplicate or incomplete responses, Policy already found from national society.
**Search**	No language limits<br>No date limits	Conference abstracts where it was not possible to obtain further information

Supplementary Table S3: Study characteristics and data

Country	Screening type	Policy Level	Data source	Estimated % Coverage
		**Developed Regions**		
**High Income**				
Australia(1)	microbiological and risk-based	national	National Society	80
Austria(2)	microbiological and risk-based	national	National Society	80
Belgium(3)	microbiological and risk-based	national	National Society	80
Canada(4)	microbiological and risk-based	national	National Society	80
Croatia(5)	risk-based	national	National Society	40
Czech Republic(6)	microbiological and risk-based	national	National Society	80
Denmark(7)	risk-based	national	National Society	60
Finland(8)	risk-based	national	National Society	60
France(9)	microbiological and risk-based	national	National Society	80
Germany(2)	microbiological and risk-based	national	National Society	60
Hungary(10)	microbiological and risk-based	national	National Society	80
Iceland(11)	risk-based	national	Online survey	60
Ireland(12)	risk-based	national	National Society	40
Israel(13)	risk-based	national	National Society	40
Italy(14)	microbiological and risk-based	national	National Society	60
Japan(15)	microbiological and risk-based	national	National Society	80
Latvia(16)	microbiological and risk-based	national	National Society	80
Lithuania	microbiological and risk-based	national	Online survey	80
Netherlands(17)	risk-based	national	National Society	60
New Zealand(1)	risk-based	national	National Society	60
Norway(11)	risk-based	national	National Society	60

Poland(18)	microbiological and risk-based	national	National Society	80
Portugal(19)	microbiological and risk-based	national	National Society	80
Slovakia(20)	microbiological and risk-based	national	National Society	80
Slovenia	microbiological and risk-based	national	National Society	80
Spain(21)	microbiological and risk-based	national	National Society	80
Sweden(11)	risk-based	national	National Society	60
Switzerland(22)	microbiological and risk-based	national	National Society	80
United Kingdom(12)	risk-based	national	National Society	60
United States of America(23)	microbiological and risk-based	national	National Society	80
**Upper-Middle Income**				
Bulgaria(24)	microbiological and risk-based	national	National Society	60
Montenegro	microbiological and risk-based	national	Online survey	60
**Lower-Middle Income**				
Ukraine	risk-based	hospital	Online survey	20
		**Asia**		
**High Income**				
Hong Kong(25)	microbiological and risk-based	national	National Society	80
Kuwait	risk-based	hospital	Online survey	20
Oman(26)	microbiological and risk-based	hospital	Literature review	20
Qatar	none	national	Literature review	0
Saudi Arabia(27)	risk-based	hospital	Literature review	20
Singapore(28)	microbiological and risk-based	national	National Society	80
South Korea(12)	risk-based	national	National Society	60
Taiwan(29)	microbiological and risk-based	national	National Society	80

United Arab Emirates	none	national	Literature review	0
**Upper-Middle Income**				
China	none	national	Online survey	0
Iran(30)	microbiological and risk-based	hospital	Literature review	20
Iraq	none	national	Literature review	0
Jordan	microbiological and risk-based	hospital	Online survey	20
Lebanon(31)	risk-based	hospital	Literature review	20
Malaysia	none	national	Literature review	0
Mongolia	none	national	Online survey	0
Thailand(32)	risk-based	hospital	Literature review	20
Turkey	none	national	National Society	0
**Lower-Middle Income**				
Bangladesh(33)	risk-based	hospital	Literature review	20
India(34)	none/risk based	hospital	Literature review	20
Myanmar	none	national	Online survey	0
Pakistan	none	national	National Society	0
Philippines(32)	risk-based	hospital	Online survey	20
Sri Lanka	none	national	National Society	0
**Low Income**				
Afghanistan*	risk-based	hospital	National Society	20
Nepal	none	national	Online survey	0
		**Latin American and the Caribbean**		
**High Income**				
Argentina(35)	microbiological and risk-based	national	National Society	60

Chile (36)	microbiological and risk-based	national	National Society	60
Trinidad and Tobago(37)	risk-based	hospital	Literature review	20
Uruguay(38)	microbiological and risk-based	national	National Society	80
**Upper-Middle Income**				
Brazil(39)	microbiological and risk-based	national	National Society	60
Costa Rica(40)	microbiological and risk-based	hospital	National Society	20
Grenada	microbiological and risk-based	national	Online survey	60
Mexico	microbiological and risk-based	national	Online survey	60
Panama(41)	risk-based	national	Literature review	25
**Lower-Middle Income**				
El Salvador	none	national	National Society	0
Guatemala	none	national	National Society	0
**Low Income**				
Haiti	none	national	Online survey	0
		**Africa**		
**Upper-Middle Income**				
Libya	none	national	National Society	0
Mauritius	risk-based	hospital	Online survey	20
South Africa(42)	risk-based	national	National Society	20
Tunisia	risk-based	hospital	Online survey	20
**Lower-Middle Income**				
Cameroon	none	national	National Society	0
Egypt	none	national	Online survey	0
Ghana	none	national	Literature review	0

Ivory Coast	none	national	National Society	0
Kenya(43)	risk-based	national	National Society	0
Nigeria	none	national	National Society	0
Senegal	none	national	National Society	0
**Low Income**				
Benin	none	national	National Society	0
Burkina Faso	none	national	National Society	0
Eritrea	none	national	National Society	0
Ethiopia	none	national	National Society	0
Gambia	none	national	Online survey	0
Guinea	none	national	National Society	0
Liberia	none	national	Online survey	0
Malawi	none	national	National Society	0
Mozambique	none	national	Literature review	0
Niger	none	national	National Society	0
Uganda	none	national	National Society	0
United Republic of Tanzania(44)	Risk-based	hospital	Literature review	20

* data from Medecins sans Frontieres.

References

1. Diseases ASfI. Management of preinatal infections 2014 [Available from: https://www.ranzcog.edu.au/RANZCOG_SITE/media/RANZCOG-MEDIA/Women's Health/Statement and guidelines/Clinical-Obstetrics/ASID-Mgnt-Perinatal-Infections-2014.pdf?ext=.pdf.
2. Berufsverband der Frauenärzte e.V. (BVF) BDfKeV, (BVDfK) DGfGuGD, Deutsche Gesellschaft für Hygiene und Mikrobiologie (DGHM), Deutsche Gesellschaft für Pädiatrische , Infektiologie (DGPI) DGfPMDuGfNupIG, Österreichische Gesellschaft für Gynäkologie und Geburtshilfe. Prophylaxe der Neugeborenensepsis frühe Form durch Streptokokken der Gruppe B 2016 [Available from: http://www.oeggg.at/fileadmin/user_upload/FAG_Streptok-AWMF_Leitlinie.pdf.
3. Council BH. PREVENTION OF PERINATAL GROUP B STREPTOCOCCAL INFECTIONS , Guidelines from the Belgian Health Council 2003 [Available from: https://www.health.belgium.be/sites/default/files/uploads/fields/fpshealth_theme_file/4448391/Prevention of perinatal group B streptococcal infections %28April 2003%29 %28SHC 7721%29.pdf.
4. Deborah Money VMA, MD. SOGC Clinical Practice Guideline: The Prevention of Early-OnsetNeonatal Group B Streptococcal Disease 2013 [Available from: https://sogc.org/wp-content/uploads/2013/09/October2013-CPG298-ENG-Online_Final.pdf.
5. zdravstvo Hzzj. SCREENING NA BHSB (beta-hemolitički streptokok grupe B; Streptococcus β-haemolyticus gr. B) 2017 [Available from: https://www.hzjz.hr/?s=streptokok+grupe+B.
6. A. Měchurová VU, J. Mašata, P. Švihovec. Diagnostika a lecba streptokoku skupiny b v tehotensvi a za porodu doporuceny postup 2013 [Available from: http://www.gynultrazvuk.cz/data/clanky/6/dokumenty/p-2013-diagnostika-a-lecba-streptokoku-skupiny-b-v-tehotenstvi.pdf.
7. Vinnie Hornshøj Greve RBH, Tine Brink Henriksen,Helle Krogh Johansen,Kathrine Birch Petersen. GBS Guideline 2012 [Available from: http://gynobsguideline.dk/sandbjerg/120426 GBS guideline endelig 25 4 12.pdf.
8. Uotila J LO. Vastasyntyneen varhaisen B-ryhmän streptokokki-infektion ehkäisy 2012 [Available from: http://www.thl.fi/attachments/Infektiotaudit/Torjuntaohjeet/Vastasyntyneen_varhaisen_B_ryhman_streptokokki_infektion_ehkaisy.pdf.
9. sante ANdaedee. Prévention anténatale du risque infectieux bactérien néonatal précoce 2001 [Available from: http://www.has-sante.fr/portail/upload/docs/application/pdf/prevention_antenatale_du_risque_infectieux_bacterien_-_syn.pdf.
10. Infomed. Újszülött csecsemők B csoport streptococcus A fertőzés megelőzése 2017 [Available from: http://www.informed.hu/betegsegek/betegsegek_reszletesen/infections/bacillaris/strepto?article_hid=1157.
11. Gynaecology NfoOa. Group B streptococcus in pregnancy and delivery 2017 [Available from: http://www.nfog.org/files/guidelines/11 NGF Obst Group B Strep Hordnes.pdf.
12. Gynaecology RCoOa. Green-top Guideline No. 36 The Prevention of Early-onset Neonatal Group B Streptococcal Disease 2012 [Available from: https://www.rcog.org.uk/globalassets/documents/guidelines/gtg_36.pdf.
13. Ginsberg GM, Eidelman AI, Shinwell E, Anis E, Peyser R, Lotan Y. Should Israel screen all mothers-to-be to prevent early-onset of neonatal group B streptococcal disease? A cost-utility analysis. Isr J Health Policy Res. 2013;2(1):6.
14. salute Md. Sistema nazionale per le linee guida: Gravidanza fisiologica 2011 [Available from: http://www.snlg-iss.it/cms/files/LG_Gravidanza.pdf.
15. Gynaecology JSoOa. 感染症合併妊 2011 [Available from: http://www.jsog.or.jp/activity/pdf/kenshu_63-7.pdf.
16. Luguze L AA. Streptococcal diseases of group B, its screening efficiency and role in neonatal and gynecological practice. nt J Reprod Contracept Obstet Gynecol. 2015;4(4):934-40.

17. gynaecology Nvvo. NVOG Voorlichtingsbrochure GROEP B STREPTOKOKKEN EN ZWANGERSCHAP 2009 [Available from: http://www.nvog.nl/Sites/Files/0000000188_GROEP B STREPTOKOKKEN EN ZWANGERSCHAP.pdf.
18. Szymusik I, Kosinska-Kaczynska K, Krolik A, Skurnowicz M, Pietrzak B, Wielgos M. The usefulness of the universal culture-based screening and the efficacy of intrapartum prophylaxis of group B Streptococcus infection. J Matern Fetal Neonatal Med. 2014;27(9):968-70.
19. Secção de Neonatologia da Sociedade Portuguesa de Pediatria. Consenso Clínico Rastreio e Prevenção da Doença Perinatal causada pelo Streptococcus agalactiae

Rastreio e Prevenção da Doença Perinatal causada pelo Streptococcus agalactiae 2012 [Available from: http://www.lusoneonatologia.com/site/upload/consensos/2013-StreptoB.pdf.
20. Stillova L, Strechova Z, Matasova K, Kolarovszka H, Bodova K, Stilla J, et al. Postnatal penicillin prophylaxis of early-onset group B streptococcal infection in term newborns. A preliminary study. Biomed Pap Med Fac Univ Palacky Olomouc Czech Repub. 2007;151(1):79-83.
21. Alós Cortésa JI DA, Arribas Mirc L, Cabero Rourad L, de Cueto Lópeze M, López Sastref J, Melchor Marcosg JC, Puertas Prietoh A, de la Rosa Frailei M, Salcedo Abizandaj S, Sánchez Lunak M, Sanchez Pérezl MJ, Torrejon Cardoso R. Prevención de la infección perinatal por estreptococo del grupo B. Recomendaciones españolas. Enferm Infecc Microbiol Clin 2013;31:159-72.
22. D. Surbek AH-G, J. Seydoux, Ch. Honegger, O. Irion, G. Drack. Prophylaxe der Early onset Neugeborenensepsis durch Streptokokken der Gruppe B 2012 [Available from: http://www.sggg.ch/fileadmin/user_upload/Dokumente/3_Fachinformationen/1_Expertenbriefe/De/19_Prophylaxe_Neugeborenensepsis_Streptokokken_2012.pdf.
23. Verani JR, McGee L, Schrag SJ, Division of Bacterial Diseases NCfI, Respiratory Diseases CfDC, Prevention. Prevention of perinatal group B streptococcal disease--revised guidelines from CDC, 2010. MMWR Recomm Rep. 2010;59(RR-10):1-36.
24. Ministry of Health Center for Public Health and Analysis B. Стрептококи от група В /СГВ/ 2017 [Available from: http://www.bsobgyn.com/index.php.
25. Health fHSDo. Prevention of Neonatal Group B Streptococcus Infection 2017 [Available from: http://www.fhs.gov.hk/english/health_info/woman/478.pdf.
26. Gupta B, El Amin E. Changing patterns of blood borne sepsis in special care baby unit, khoula hospital. Oman Med J. 2010;25(2):100-3.
27. Al-Kadri HM, Bamuhair SS, Johani SM, Al-Buriki NA, Tamim HM. Maternal and neonatal risk factors for early-onset group B streptococcal disease: a case control study. Int J Womens Health. 2013;5:729-35.
28. Singapore oagso. Group B Streptococcus screening 2017 [Available from: http://www.nuhgynae.com.sg/cos/o.x?c=/wbn/pagetree&func=view&rid=1039981.
29. Taiwan Association of Obstetrics and Gynaecology [Available from: http://www.taog.org.tw/member/MBPasswd.asp.
30. Shirazi M, Abbariki E, Hafizi A, Shahbazi F, Bandari M, Dastgerdy E. The prevalence of group B streptococcus colonization in Iranian pregnant women and its subsequent outcome. Int J Fertil Steril. 2014;7(4):267-70.
31. Seoud M, Nassar AH, Zalloua P, Boghossian N, Ezeddine J, Fakhoury H, et al. Prenatal and neonatal Group B Streptococcus screening and serotyping in Lebanon: incidence and implications. Acta Obstet Gynecol Scand. 2010;89(3):399-403.
32. Villanueva-Uy M WP, Sangtawesin V, Chiu V, Tallo V, Nazaire-Bermal N, Bock H, Cunnington M, Nan C, Boudville I. THE BURDEN OF INVASIVE NEONATAL GROUP B STREPTOCOCCAL (GBS) DISEASE IN THAILAND AND THE PHILIPPINES. South East Asian Journal of tropical medicine and public health. 2015;46(4):728-37.
33. Chan GJ, Stuart EA, Zaman M, Mahmud AA, Baqui AH, Black RE. The effect of intrapartum antibiotics on early-onset neonatal sepsis in Dhaka, Bangladesh: a propensity score matched analysis. BMC Pediatr. 2014;14:104.

34. Narava S, Rajaram G, Ramadevi A, Prakash GV, Mackenzie S. Prevention of perinatal group B streptococcal infections: a review with an Indian perspective. Indian J Med Microbiol. 2014;32(1):6-12.
35. Nación MdSdl, INFANTIL DNDSM. Recomendaciones para la prevención, diagnóstico y tratamiento de la infección neonatal precoz por Estreptococo β Hemolítico del Grupo B (EGB) 2004 [Available from: http://www.msal.gob.ar/images/stories/bes/graficos/0000000318cnt-consenso-estreptococo-B-hemolitico.pdf.
36. Abarzua F, Arias A, Garcia P, Ralph C, Cerda J, Riedel I, et al. [Streptococcus agalactiae increase in resistance to erythromycin and clindamycin in vaginal-anal colonization in third quarter of pregnancy in one decade of universal screening]. Rev Chilena Infectol. 2011;28(4):334-7.
37. Orrett FA. Colonization with Group B streptococci in pregnancy and outcome of infected neonates in Trinidad. Pediatr Int. 2003;45(3):319-23.
38. Género MdSPDGdlSPNPdSdlMy. Normas de Atención a la Mujer Embarazada 2007 [Available from: https://www.sguruguay.org/documentos/msp-gssr-capitulo-normas-atencion-mujer-embarazada.pdf.
39. Almeida A AJ, Ferreira L. Consensos em Neonatologia Estreptococo ⍰ HemolÌtico do Grupo B Protocolo de Rastreio e PrevenÁ„o de DoenÁa Perinatal 2011 [Available from: http://www.lusoneonatologia.com/site/upload/File/Streptococcus B hemolitico do grupo B.pdf.
40. NM CC. STREPTOCOCCUS AGALACTIAE, DETECCIÓN Y MANEJO INTRAPARTO. REVISTA MEDICA DE COSTA RICA Y CENTROAMERICA 2016;LXXIII (618):161-4.
41. Rivera L, Saez-Llorens X, Feris-Iglesias J, Ip M, Saha S, Adrian PV, et al. Incidence and serotype distribution of invasive group B streptococcal disease in young infants: a multi-country observational study. BMC Pediatr. 2015;15:143.
42. gynaecology SASooa. SUGGESTED GUIDELINES FOR SPECIAL INVESTIGATIONS REQUESTED IN ANTE NATAL CARE OF WOMEN. 2012 [Available from: https://www.sasog.co.za/Guidelines/Index.
43. services Mophasamom. National Guidelines for quality obstetrics and perinatal care 2012 [Available from: https://www.k4health.org/sites/default/files/National Guidelines for Quality Obstetrics and Perinatal Care.pdf.
44. Joachim A, Matee MI, Massawe FA, Lyamuya EF. Maternal and neonatal colonisation of group B streptococcus at Muhimbili National Hospital in Dar es Salaam, Tanzania: prevalence, risk factors and antimicrobial resistance. BMC Public Health. 2009;9:437.
