# Supplementary material for: Intrapartum Antibiotic Chemoprophylaxis Policies for the Prevention of Group B Streptococcal Disease Worldwide: Systematic Review
Source: Clin Infect Dis. 2017 Nov 6;65(Suppl 2):S143–51. doi: 10.1093/cid/cix654 (PMC5850619; doi:10.1093/cid/cix654)
Supplement: Supplement material 2 [file cix654_suppl_supplement-material2.pdf]

Group B Streptococcus (GBS).
Ante-natal testing and intra-partum antibiotic prophylaxis for the prevention of GBS disease in newborns.

Welcome to our Survey

Group B streptococcus infection is a significant cause of mortality and morbidity in newborns world wide.

We are an international group of clinicians working on mapping the GBS disease and identifying global research gaps for a Clinical Infectious Diseases series funded by the Bill and Melinda Gates Foundation.

This survey is for any health care professionals working in child and maternal health or those with an interest in infectious diseases. The survey should take no more than 10 minutes to complete. Once you have completed the survey if you have any data that you can contribute and wish to be named we would be delighted to include you as one of a journal paper author group named on pubmed.

Thank you for taking the time to complete the survey, your input is vital to a better understanding of antibiotic use to prevent early onset GBS disease.

Group B Streptococcus (GBS).
Ante-natal testing and intra-partum antibiotic prophylaxis for the prevention of GBS disease in newborns.

* 1. Does the country you work in have a National Guideline for the screening and management of Group B streptococcus (GBS) infection in pregnancy?

○ Yes

○ No

Group B Streptococcus (GBS).
Ante-natal testing and intra-partum antibiotic prophylaxis for the prevention of GBS disease in newborns.

* 2. Are the National Guidelines easily available for you to review?

- Yes
- No

* 3. Does the national guideline support GBS screening for all women as part of their routine care during pregnancy?

- Yes
- No

Group B Streptococcus (GBS).
Ante-natal testing and intra-partum antibiotic prophylaxis for the prevention of GBS disease in newborns.

* 4. Does your guideline support opportunistic testing of pregnant women for GBS?

- Yes
- No

Group B Streptococcus (GBS).
Ante-natal testing and intra-partum antibiotic prophylaxis for the prevention of GBS disease in newborns.

* 5. At what gestation does your guideline suggest you screen pregnant women for GBS?

* 6. Which swabs does the national guideline recommend you take?

- ◯ Vaginal
- ◯ Rectal
- ◯ Vaginal and Rectal

* 7. Does the guideline recommend either microbiology or PCR for swab analysis?

- ◯ Microbiology
- ◯ PCR
- ◯ Either
- ◯ Not specified

* 8. Does your national guideline recommend intrapartum antibiotic prophylaxis for women who test positive for GBS?

- ◯ Yes
- ◯ No

* 9. Which antibiotics does the National Guideline recommend that you use for prophylaxis against GBS in women who are not allergic to penicillin?

- ◯ Penicillin G
- ◯ Ampicillin
- ◯ Benzypenicillin
- ◯ Other (please specify)

* 10. Which antibiotics does your National Guideline recommend that your use for prophylaxis against GBS in women who are allergic to penicillin?

- ◯ Clindamycin
- ◯ Cefazolin
- ◯ Vancomycin
- ◯ Other (please specify)

* 11. Are these antibiotics always readily available for women in labour?

- Yes
- No

* 12. Do you have a local/hospital guideline for the management of GBS in pregnancy?

- Yes
- No

Group B Streptococcus (GBS).
Ante-natal testing and intra-partum antibiotic prophylaxis for the prevention of GBS disease in newborns.

* 13. Is the local/hospital guideline the same as the national guideline?

- Yes
- No
- No, because we don't have a national guideline.

Group B Streptococcus (GBS).
Ante-natal testing and intra-partum antibiotic prophylaxis for the prevention of GBS disease in newborns.

* 14. Does the hospital guideline recommend GBS screening for all pregnant women?

- Yes
- No

Group B Streptococcus (GBS).
Ante-natal testing and intra-partum antibiotic prophylaxis for the prevention of GBS disease in newborns.

* 15. At what gestation does your hospital guideline recommend screening for GBS in pregnancy?

* 16. Which swabs does the guideline recommend you take?

- Vaginal
- Rectal
- Vaginal and Rectal

* 17. How are the swabs analyzed?

- Microbiology
- PCR

* 18. Does your hospital guideline recommend intrapartum antibiotic prophylaxis for women who test positive for GBS?

- Yes
- No

* 19. Which antibiotics does your hospital guideline recommend that you use for prophylaxis against GBS in women who are not allergic to penicillin?

- Penicillin G
- Ampicillin
- Benzypenicillin

* 20. Which antibiotics does your hospital guideline recommend that your use for prophylaxis against GBS in women who are allergic to penicillin?

- Clindamycin
- Cefazolin
- Vancomycin

* 21. Are antibiotics always readily available for women in labour?

- Yes
- No

* 22. Are antibiotics only available with a prescription from a medical doctor in your country?

- ◯ Yes, only medical practitioners can prescribe antibiotics
- ◯ No, antibiotics are available from a wide range of healthcare practitioners
- ◯ No, antibiotics can be brought without prescription

* 23. Does your hospital screen women in preterm labour for GBS infection?

- ◯ Yes
- ◯ No

* 24. Does your hospital screen women women with preterm spontaneous rupture of membranes for GBS infection?

- ◯ Yes
- ◯ No

* 25. Does your hospital routinely collect data on the number of women who test positive for GBS during routine screening in pregnancy?

- ◯ Yes
- ◯ No

Group B Streptococcus (GBS).
Ante-natal testing and intra-partum antibiotic prophylaxis for the prevention of GBS disease in newborns.

* 26. What is the rate of GBS colonization in pregnant women in your hospital? (approximately per year)

____

* 27. Does your hospital routinely collect data on the number of babies who develop GBS sepsis in your hospital?

- ◯ Yes
- ◯ No

Group B Streptococcus (GBS).
Ante-natal testing and intra-partum antibiotic prophylaxis for the prevention of GBS disease in newborns.

* 28. What is the rate of GBS disease in newborns in your hospital? (approximately per year)

[ ]

* 29. Is any of this data published?

- ◯ Yes
- ◯ No

Group B Streptococcus (GBS).
Ante-natal testing and intra-partum antibiotic prophylaxis for the prevention of GBS disease in newborns.

30. Where can the data be accessed?

[ ]

31. Do you have any unpublished data that you could share with us?

- ◯ Yes - I will add my contact details at the end of the survey
- ◯ No

32. One of our papers will look specifically at GBS-disease in pregnancy. If you have any relevant unpublished data that you would be willing to share with us in an anonymised, aggregated form please get in touch with Dr. Jennifer Hall (Jennifer.hall@ucl.ac.uk) to discuss this. Anyone contributing such data will be eligible to be part of the investigator group.’

[ ]

33. What is the name of your hospital?

[ ]

34. Is your patient population

- ◯ Mostly urban
- ◯ Mostly rural
- ◯ A mix of both

* 35. Approximately how many babies does your hospital deliver each year?

[ ]

* 36. Which country do you work in?

[ ]

* 37. Does you country routinely collect data on GBS colonization in pregnant women?

- ◯ Yes
- ◯ No
- ◯ Don't know

Group B Streptococcus (GBS).
Ante-natal testing and intra-partum antibiotic prophylaxis for the prevention of GBS disease in newborns.

* 38. What is the approximate annual rate GBS colonization in pregnant women in your country? (if you are not sure just put "don't know".)

[ ]

* 39. Does you country routinely collect data of GBS disease in newborns?

- ◯ Yes
- ◯ No
- ◯ Don't know

Group B Streptococcus (GBS).
Ante-natal testing and intra-partum antibiotic prophylaxis for the prevention of GBS disease in newborns.

* 40. What is the annual rate (approximately) of GBS disease in newborns in your country?

* 41. Are you a?

- Consultant/Attending Obstetrician
- Trainee/Resident Obstetrician
- Consultant/Attending Pediatrician/Neonatologist
- Trainee/Resident pediatrician/neotaologist
- Midwife
- Other (please specify)

42. If you would like be a named author in the group please let us have your contact details;

Name

Company

Address 2

City/Town

State/Province -- select state --

ZIP/Postal Code

Country

Email Address

43. If there is any other information you would like to tell us about your GBS screening program please enter it here.

44. Thank you again for taking the time to complete this survey.
If you have any further questions about this survey or GBS prophylaxis you can contact us at ScreeningGBS@gmail.com
